# Muscle density is an independent risk factor of second hip fracture: a prospective cohort study

**DOI:** 10.1002/jcsm.12996

**Published:** 2022-04-15

**Authors:** Ling Wang, Lu Yin, Minghui Yang, Yufeng Ge, Yandong Liu, Yongbin Su, Zhe Guo, Dong Yan, Zhengyang Xu, Pengju Huang, Jian Geng, Xingli Liu, Gang Wang, Glen M. Blake, Weiming Cao, Bo He, Liang Lyu, Xiaoguang Cheng, Xinbao Wu, Lihong Jiang, Annegreet Vlug, Klaus Engelke

**Affiliations:** ^1^ Department of Radiology Beijing Jishuitan Hospital Beijing China; ^2^ Department of Radiology The First People's Hospital of Yunnan Province Kunming China; ^3^ Medical Research & Biometrics Center National Center for Cardiovascular Disease Beijing China; ^4^ Departments of Traumatic Orthopedics Beijing Jishuitan Hospital Beijing China; ^5^ Department of Radiology the First Medical Center of Chinese PLA General Hospital Beijing China; ^6^ School of Biomedical Engineering & Imaging Sciences, King's College London St Thomas' Hospital London UK; ^7^ Health Commission of Yunnan Province Kunming China; ^8^ Department of Radiology The First Affiliated Hospital of Kunming Medical University Kunming China; ^9^ The First People's Hospital of Yunnan Province Kunming China; ^10^ Center for Bone Quality, Department of Internal Medicine Leiden University Medical Center Leiden The Netherlands; ^11^ Department of Medicine 3 FAU University Erlangen‐Nürnberg and Universitätsklinikum Erlangen Erlangen Germany; ^12^ Institute of Medical Physics University of Erlangen Erlangen Germany

**Keywords:** Second hip fracture, Muscle density, Muscle size, Bone mineral density

## Abstract

**Background:**

Patients with a first hip fracture are at high risk of fracturing their other hip. Despite this, preventive therapy is often not given. Because little is known about specific risk factors of a second hip fracture, we investigated the association with areal bone mineral density (aBMD), muscle size, and density. We also investigated whether muscle parameters predict the risk of a contralateral fracture independently of aBMD.

**Methods:**

Three groups were included, one without hip fracture (a subcohort of the China Action on Spine and Hip Status study), one with a first, and one with a second hip fracture. Subjects with fractures were recruited from the longitudinal Chinese Second Hip Fracture Evaluation (CSHFE). Computed tomography scans of CSHFE patients, which were obtained immediately following their first fracture, were used to measure cross‐sectional area and density of the gluteus maximus (G.MaxM) and gluteus medius and minimus (G.Med/MinM) muscles. Computed tomography X‐ray absorptiometry was used to measure aBMD of the contralateral femur. Median follow‐up time to second fracture was 4.5 years. Cox proportional hazards models were used to compute hazard ratios (HR) of second hip fracture risk in subjects with a first hip fracture. Multivariate logistic regressions were used to compare odds ratios (OR) for the risk of a first and second hip fracture.

**Results:**

Three hundred and one participants (68.4 ± 6.1 years, 64% female) without and 302 participants (74.6 ± 9.9 years, 71% female) with a first hip fracture were included in the analysis. Among the latter, 45 (79.2 ± 7.1 years) sustained a second hip fracture. ORs for first hip fracture were significant for aBMD and muscle size and density. ORs for a second fracture were smaller by a factor of 3 to 4 and no longer significant for femoral neck (FN) aBMD. HRs for predicting second hip fracture confirmed the results. G.Med/MinM density (HR, 1.68; CI, 1.20–2.35) and intertrochanter aBMD (HR, 1.62; CI, 1.13–2.31) were the most significant. FN aBMD was not significant. G.Med/MinM density remained significant for predicting second hip fracture after adjustment for FN (HR, 1.66; Cl, 1.18–2.30) or total hip aBMD (HR, 1.50; 95% Cl, 1.04–2.15).

**Conclusions:**

Density of the G.Med/MinM muscle is an aBMD independent predictor of the risk of second hip fracture. Intertrochanteric aBMD is a better predictor of second hip fracture than FN and total hip aBMD. These results may trigger a paradigm shift in the assessment of second hip fracture risk and prevention strategies.

## Introduction

Worldwide, hip fractures are an important cause of morbidity and mortality that severely reduce quality of life and pose a major economic health burden.^1^, ^2^, ^3^ Hip fractures have a substantial impact on mobility and physical functioning with only one‐third to one‐half of hip fracture patients regaining their prior ambulatory function.^4^, ^5^, ^6^ In addition, suffering a first hip fracture is associated with a two to four times higher risk of suffering a second fracture of the opposite hip.^7^, ^8^ Further, a second hip fracture has an even poorer prognosis than the first.^9^, ^10^ Therefore, a thorough risk assessment following a first hip fracture should be performed as a basis for a detailed intervention, for example, to decide between antiresorptive and anabolic therapy, to optimize physical exercise, or to apply local osteo‐enhancement procedures currently undergoing clinical validation.^11^

Standard procedures of fracture risk assessment include scoring systems using age, prevalent fractures, bone mineral density (BMD), and clinical risk factors as variables.^12^ However, there is little scientific evidence guiding the adequate prediction of second hip fracture risk. Areal BMD (aBMD) assessed by dual‐energy X‐ray absorptiometry is an established parameter for the prediction of hip fracture risk. However, risk of a second fracture is significantly elevated for patients with hip or vertebral fractures independent of BMD.^13^ Thus, aBMD measurements of the hip may have limited value for predicting the risk of a second hip fracture.

Independent of BMD, hip fracture risk is related to muscle weakness that contributes to the incidence of falls.^14^ Reduction in muscle performance is strongly associated with increases in muscle fatty infiltration. Therefore, previous computed tomography (CT) studies have targeted not only muscle size but also muscle composition as potential contributors to hip fracture risk.^15^, ^16^, ^17^, ^18^ However, the performance of muscle assessments for prediction of risk of a second hip fracture is still unknown.

In this study, we first aimed to investigate whether BMD, hip structural geometry, and muscle size and density parameters predict risk of a second hip fracture in patients suffering a first fracture, and second whether muscle parameters do so independently of femoral neck (FN) or total hip (TH) aBMD. A third aim was to compare associations of muscle parameters and BMD with hip fracture risk between subjects with first and second hip fracture. We hypothesized that muscle density was a stronger predictor of the risk of a second hip fracture than bone parameters or muscle size. We further hypothesized that muscle density was lowest in subjects with second hip fracture and highest in controls without hip fracture.

## Methods

### Study design and participants

Three groups were included in the study, a control group without hip fracture, a group with first hip fracture and a group with second hip fractures. The control group was a subcohort of 301 participants (mean age 68 years, 64% female) of the China Action on Spine and Hip Status study (CASH, Clinical Trials.gov Identifier: NCT01758770),^19^ a multi‐centre epidemiological study focused on quantitative CT and dual‐energy X‐ray absorptiometry to determine the prevalence of osteoporosis and osteoporotic spinal fractures in an elderly Chinese population. The control group was recruited in the neighbourhood of Beijing Jishuitan Hospital. Details were described previously.^18^

All subjects with hip fractures were recruited for the Chinese Second Hip Fracture Evaluation (CSHFE, Clinical Trials.gov Identifier: NCT03461237), a prospective longitudinal study to evaluate the risk of a second hip fracture in patients with a first hip fracture.^20^ A total of 668 subjects with low‐energy hip fractures admitted to the Beijing Jishuitan Hospital emergency department of orthopaedic trauma between May 2015 and June 2016 were recruited for this study. The clinical approach has been described previously.^20^ The present analysis used CT scans from subjects obtained immediately (<48 h) after the first fragility hip fracture (baseline visit). Patients enrolled in the CSHFE study were followed up for a median time of 4.5 years (from 2015–2016 to 2019–2020).

Inclusion and exclusion criteria for all three groups were similar to those described by Su *et al*.^20^ In brief, only fully ambulatory, community‐dwelling Chinese Han adults were included. Exclusion criteria were inability to sit and stand independently, inability to walk with or without an assistive device, or pain that prevented testing. Further exclusion criteria were stroke, neurologic disorders, metabolic diseases, rheumatic diseases, heart failure, severe chronic obstructive pulmonary disease and coagulation disorders, and other diseases that limited function. All hip fractures resulted from low‐energy injury. Thus, only falls from standing or sitting height were considered as cause for frailty hip fracture.

For the CSHFE study, but not for CASH, that is, for the fracture but not for the control group, orthopaedists in the emergency room assessed the mobility of patients prior to the first hip fracture using the Parker Mobility Score. After 4.5 years, patients were followed up by orthopaedists by telephone for the potential incidence of a second hip fracture and/or death. The Parker Mobility Score was determined for the 3 months prior to a second hip fracture or death. If the patients neither had died nor had suffered from a second hip fracture, the orthopaedists also assessed mobility within 3 months prior to the telephone interview. Patients with a Parker Mobility Score < 3 (mobility assessment) after surgery or before death were excluded.

The study was approved by the ethics committee of Beijing Jishuitan Hospital. Informed consent was obtained from each participant.

### Muscle density and bone density assessments

Spiral CT imaging of the hip was performed for all study participants using two Toshiba Aquilion CT scanners (Toshiba Medical Systems Division, Tokyo, Japan). Scans were acquired in the supine position from the top of the acetabulum to 3 cm below the lesser trochanter (TR) and included both legs. Scan parameters were 120 kVp, 125 mAs, 50 cm field of view, 512 × 512matrix, and 1 mm reconstructed slice thickness.

Cross‐sectional area and density were measured of the gluteus maximus (G.max) at the level of the greater TR and of the gluteus medius and minimus (G.med/min) muscle at the level of the third sacral vertebra (S3) (Supporting Information, *Figure* *S1*). In subjects with hip fracture, the non‐fractured hip was analysed. If the CT scan did not cover the S3 level, the muscle density and area of the medius and minimus muscle were measured at the S4 or S5 levels. A previous study showed that there was no significant difference (0.8 Hounsfield unit) in muscle density between the S3 and non‐S3 levels.^18^ Difference in muscle area between the S3 and non‐S3 levels was not significant either. However, the absolute difference of about 4.1 cm^2^ accounted for a bias of up to 10% compared with the mean area at the S3 level. We therefore did not include gluteus medius and minimus muscle cross‐sectional area in the analysis.

OsiriX software (Lite Version 10.0.2, Pixmeo, Geneva, Switzerland) was used for the analysis. The muscle measurements procedure and precision have been previously reported.^21^

Areal BMD (aBMD, g/cm^2^) of the FN, TR, intertrochanter (IT), and TH was calculated from the hip CT scans using the computed tomography X‐ray absorptiometry technique (Version 4.2.3, Mindways Inc). Structural variables were derived using the Bone Investigation Toolkit (Mindways Software Inc.) as previously described by Wang *et al*.^22^ The Medical Image Analysis Framework option Femur (Version 7.1.0 MRH) was used to measure three‐dimensional femoral neck cortical thickness.

### Parker Mobility Score

The Parker Mobility Score is a valid and reliable measurement for the assessment of mobility.^23^ Parker Mobility Scores were assessed prior to the first hip fracture (within 3 months), prior to second fracture (within 3 months), and prior to death (within 3 months) and prior to the telephone interview for those patients without second hip fracture. The ability to move around the house, to go out of the house, and to go shopping were scored as: without difficulty (3 points), with an aid such as a walking stick (2 points) with help from another person (1 point), or impossible (0 points). Thus, the Parker Mobility Score ranges from 0 to 9.

### Data collection

Demographic and anthropometric assessments included age, sex, and body mass index. Health‐related data included blood pressure, hypertension, previous fracture, osteoarthritis, coronary heart disease, type 2 diabetes mellitus (T2DM), and antiosteoporosis treatment. The treatment of osteoporosis was defined as taking either a bisphosphonate or teriparatide.

### Statistical analysis

In this study, a prospective analysis was conducted to compare the gluteal muscle size and CT density for the prediction of a second hip fracture. For this analysis, the two groups with fractures were used. In addition, a cross‐sectional analysis was performed to compare associations of the gluteal muscle size and CT density between first and second hip fracture. For this analysis, all three study groups as described in Study design and participants section were used.

Continuous variables were analysed using two‐sample Wilcoxon tests and are reported as mean ± standard deviation. Categorical variables were analysed using *χ* ^2^ tests and are presented as numbers and percentages.

For the prospective analysis of second hip fracture risk, traditional Cox proportional hazards models (HR) were used. The 28 death events were regarded as censored data. The cumulative incidence of second hip fracture was also calculated using Kaplan–Meier survival analyses and illustrated using sex‐specific median values of various muscle and bone parameters as cut‐points. In addition, competing risk analyses using cause‐specific hazard models were conducted using death events occurring in the absence of second fracture events as competing risks. For this analysis, study subjects of the two fracture groups were divided into death, second hip fracture, and no second hip fracture subgroups. Fine–Gray models were conducted to analyse the competing risks as sensitivity analyses.^24^ Age, sex, type 2 diabetes, and Parker Mobility Scores obtained prior to second fracture were used as covariates.

The cross‐sectional analysis was applied to compare the strength of associations of bone and muscle parameters with first and second hip fracture. The area under the receiver operating characteristic curve (AUC) with 95% confidence intervals (CI) was computed using multivariate logistic regressions. Hosmer–Lemeshow tests were used to evaluate model robustness by calculating the Pearson *χ* ^2^ statistic from the table of observed and expected frequencies. If the model fits well the test result is non‐significant.

The Statistical Analysis System (SAS 9.4 for Windows; SAS Institute Inc., Cary, NC) was used for all statistical analyses.

## Results

### Study sample characteristics

**Figure 1:**
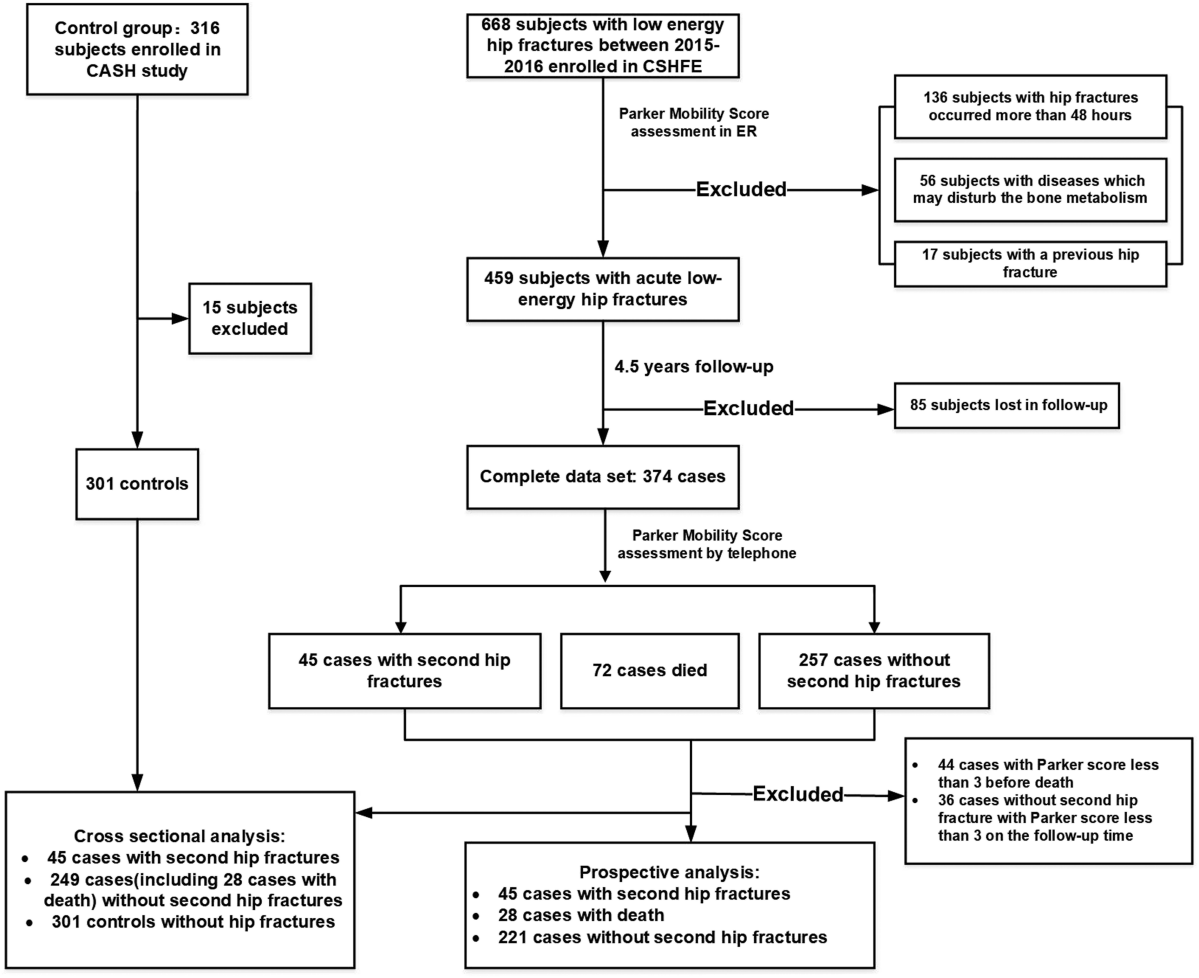
Flow chart of participant selection for the study.

*Table* *1* shows the baseline characteristics of the study population and *Figure* *1* its selection. The control group consisted of 301 subjects without follow‐up visits. After 4.5 years of follow‐up, 374 subjects of the two groups with hip fracture had completed the study (*n* = 302) or had died (*n* = 72). Forty‐four patients who died and 36 cases without a second hip fracture had Parker Mobility Scores less than 3 at follow‐up and were excluded. Of the 302 survivors, 45 had sustained a second hip fracture (20 FN and 25 trochanteric fractures) (*Figure* *1*). In the group without second hip fracture, 133 subjects had sustained a FN and 88 a TR fracture at baseline (*Figure* *S2*). The subjects suffering a second fracture (mean age: 79.2 ± 7.1 years) or death (80.6 ± 8.5 years) were older than the surviving patients without second fracture (72.6 ± 9.8 years). Parker Mobility Scores were not significantly different between the two fracture groups at baseline (i.e. prior to first hip fracture), while the patients surviving without second hip fracture had significantly higher Parker Mobility Scores than those deceased or suffering a second hip fracture.

**Table 1:** General characteristics

Characteristics (mean ± standard deviance)	Hip fracture patients					Controls (6)	*P* value^a^		
	Died	Second hip fracture	Non‐second hip fracture	Followed	Lost				
	(1)	(2)	(3)	(4) = (1) + (2) + (3)	(5)		(2) vs. (1) + (3)	(4) vs. (5)	(4) vs. (6)
*Sample size*	*28*	*45*	*221*	*294*	*85*	*301*	*—*		*—*
Age (years)	80.57 ± 8.48	79.23 ± 7.10	72.60 ± 9.84	74.37 ± 9.83	76.74 ± 7.78	68.36 ± 6.14	<0.01	0.13	<0.01
Male sex, % (*n*)	35.71 (10)	22.22 (10)	30.77 (68)	29.93 (88)	32.94 (28)	35.55 (107)	0.41	0.60	0.14
HA, % (*n*)	21.43 (6)	40.00 (18)	36.65 (81)	35.71 (105)	—	—	0.23	*—*	*—*
FN fractures, % (n)	35.71 (10)	44.44 (20)	60.18 (133)	55.44 (163)	60.49 (49)	—	0.11	0.42	*—*
Antiosteoporosis treatment, % (*n*)	21.43 (6)	20.00 (9)	12.22 (27)	14.29 (42)	—	—	0.21	*—*	*—*
Height (cm)	161.64 ± 6.74	160.41 ± 6.47	162.35 ± 9.45	162.03 ± 8.88	163.04 ± 7.83	162.47 ± 7.51	0.14	0.36	<0.01
Weight (kg)	58.38 ± 11.78	57.35 ± 15.57	61.59 ± 12.83	60.71 ± 13.15	58.85 ± 10.22	66.77 ± 10.00	0.37	0.13	0.66
BMI (kg/m^2^)	22.23 ± 3.75	22.18 ± 5.50	23.42 ± 5.16	23.14 ± 5.09	22.12 ± 3.35	25.24 ± 2.92	0.56	0.03	<0.01
Parker Mobility Score 1	8.32 ± 1.02	8.49 ± 1.10	8.73 ± 0.80	8.65 ± 0.88	—	—	0.32	*—*	*—*
Parker Mobility Score 2	6.68 ± 1.59	6.57 ± 2.55	7.95 ± 1.37	7.63 ± 1.71	—	—	<0.01	*—*	*—*
SBP (mmHg)	146.59 ± 20.4	145.61 ± 20.49	142.42 ± 20.25	143.07 ± 20.26	142.31 ± 18.63	126.51 ± 8.64	0.39	0.76	<0.01
DBP (mmHg)	79.26 ± 12.74	76.67 ± 10.57	76.54 ± 12.10	76.83 ± 12.05	75.26 ± 11.86	74.10 ± 7.56	0.89	0.47	0.01
Hypertension, % (*n*)	14.29 (4)	28.89 (13)	20.36 (45)	21.09 (62)	21.18 (62)	—	0.16	0.99	—
T2DM, % (*n*)	64.29 (18)	24.44 (11)	48.87 (108)	46.6 (137)	25.88 (22)	37.9 (114)	<0.01	<0.01	0.03
History of CHD, % (*n*)	3.57 (1)	2.22 (1)	4.98 (11)	4.42 (1)	9.41 (8)	—	0.44	0.08	—
Previous fractures, % (*n*)	14.29 (4)	17.78 (8)	21.72 (48)	20.41 (60)	27.06 (23)	21.4 (51)	0.63	0.19	0.28
OA, % (*n*)	10.71 (3)	4.44 (2)	12.22 (27)	10.88 (32)	16.47 (14)	—	0.13	0.16	—
G.MaxM area (cm^2^)	27.84 ± 6.53	29.16 ± 6.04	33.85 ± 8.46	32.56 ± 8.26	—	39.36 ± 7.43	<0.01	*—*	<0.01
G.MaxM density (HU)	20.73 ± 7.84	20.50 ± 7.77	25.02 ± 6.95	23.92 ± 7.39	—	33.45 ± 6.59	<0.01	*—*	<0.01
G.Med/MinM density (HU)	29.06 ± 6.44	27.52 ± 5.29	31.97 ± 5.83	31.02 ± 6.04	—	42.35 ± 4.39	<0.01	*—*	<0.01
FN CortThick (mm)	1.58 ± 0.25	1.56 ± 0.38	1.55 ± 0.37	1.55 ± 0.36	—	1.84 ± 0.28	0.73	*—*	<0.01
TH aBMD (g/cm^2^)	0.57 ± 0.13	0.53 ± 0.11	0.61 ± 0.13	0.59 ± 0.13	0.56 ± 0.12	0.80 ± 0.16	<0.01	0.04	<0.01
FN aBMD (g/cm^2^)	0.45 ± 0.09	0.47 ± 0.13	0.51 ± 0.11	0.50 ± 0.11	0.46 ± 0.09	0.68 ± 0.14	0.04	0.01	<0.01
TR aBMD (g/cm^2^)	0.38 ± 0.11	0.35 ± 0.09	0.41 ± 0.10	0.40 ± 0.10	0.37 ± 0.10	0.56 ± 0.13	<0.01	0.12	<0.01
IT aBMD (g/cm^2^)	0.70 ± 0.17	0.64 ± 0.13	0.74 ± 0.15	0.73 ± 0.16	0.69 ± 0.15	0.98 ± 0.19	<0.01	0.06	<0.01
bCSA (cm^2^)	1.53 ± 0.31	1.48 ± 0.30	1.66 ± 0.37	1.62 ± 0.36	1.53 ± 0.35	2.07 ± 0.46	<0.01	0.04	<0.01
ACT (cm)	0.12 ± 0.04	0.11 ± 0.02	0.12 ± 0.02	0.12 ± 0.03	0.11 ± 0.02	0.16 ± 0.04	0.02	<0.01	<0.01
CSMI (cm^4^)	1.48 ± 0.48	1.37 ± 0.53	1.61 ± 0.77	1.56 ± 0.72	1.52 ± 0.83	1.72 ± 0.67	0.06	0.44	<0.01
Z (cm^3^)	0.84 ± 0.25	0.79 ± 0.29	0.90 ± 0.37	0.88 ± 0.35	0.81 ± 0.33	1.03 ± 0.36	0.05	0.09	<0.01
BR	16.26 ± 5.57	16.36 ± 4.72	15.00 ± 3.65	15.33 ± 4.06	17.44 ± 4.60	11.00 ± 3.31	0.06	<0.01	<0.01

aBMD, areal bone mineral density; ACT, average cortical thickness; bCSA, bone cross‐sectional area; BMI, body mass index; BR, buckling ratio; CHD, coronary heart diseases; CortThick, cortical thickness; CSMI, cross‐sectional moment of inertia; DBP, diastolic blood pressure; FN, femoral neck; G.MaxM, gluteus maximus muscle; G.Med/MinM, gluteus medius and minimus muscle; HA, hip arthroplasty (including total hip arthroplasty and hemiarthroplasty); HU, Hounsfield unit; IT, intertrochanter; OA, osteoarthritis; SBP, systolic blood pressure; T2DM, type 2 diabetes; TH, total hip; TR, trochanter; Z, section modulus.

Parker Mobility Score 1: assessment obtained prior to first hip fracture surgery; Parker Mobility Score 2: refracture group: assessment within 3 months prior to second hip fracture; death group: assessment within 3 months prior to death; group without second fracture: assessment of mobility prior to follow‐up visit.

^a^ *P* value was obtained using *χ* ^2^ tests for categorical variables and two‐sample Wilcoxon tests for continuous variables.

As shown in *Table* *1*, compared with the death and second fracture groups, most muscle and aBMD parameters were highest in surviving patients without second hip fracture, except for femoral neck cortical thickness. In the controls, all muscle and BMD variables were significantly higher than in hip fracture patients.

### Muscle and bone: prediction of second hip fracture

**Figure 2:**
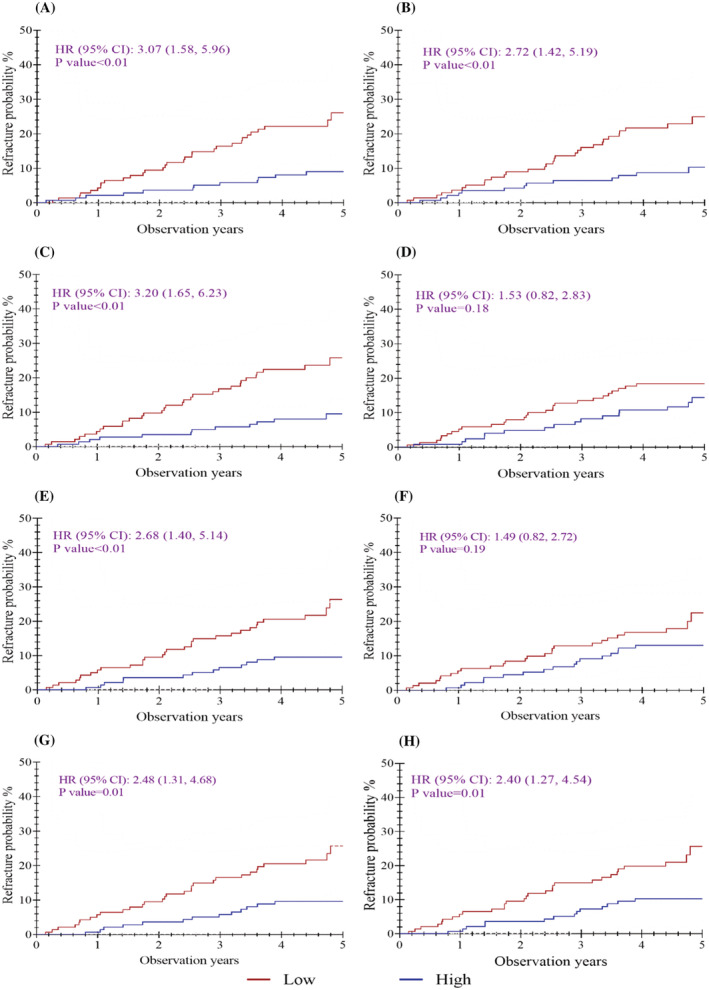
Kaplan–Meier curves for probability of second hip fracture by low vs. high parameter values using median as cut‐points. (*A*) G.MaxM area, (*B*) G.MaxM density, (*C*) G.Med/MinM density, (*D*) FN CortThick, (*E*) TH aBMD, (*F*) FN aBMD, (*G*) TR aBMD, and (*H*) IT aBMD. CI, confidence interval; HR, hazard ratio.

*Figure* *2* illustrates the cumulative incidence of second hip fracture using Kaplan–Meier survival curves. For each parameter shown in the plots, the high and low risk groups were differentiated using sex‐specific median values of the specific parameter. Higher second hip fracture probabilities were observed for lower G.MaxM area; lower G.MaxM and G.Med/MinM density; and lower aBMD of TH, TR, and IT. Second hip fracture probabilities did not significantly differ between high and low risk groups for FN cortical thickness, FN aBMD, and all structural variables (*P* > 0.05).

With the exception of G.MaxM area, the significance of these results was confirmed using traditional Cox proportional hazards models after adjustments for age, sex, T2DM, and Parker Mobility Scores obtained prior to surgery (*Table* *2*). Also, HR of TR aBMD was no longer significant after the competing risk analysis. There were no significant differences between adjusted HRs from Cox proportional hazards and Fine–Gay models. *Table* *S1* shows the same analysis as *Table* *2* but without excluding subjects because of poor mobility (Parker Mobility Score < 3). *Table* *S2* also shows the same analysis as *Table* *2* but further adjusted for health‐related data. Both results were consistent with those in *Table* *2*.

**Table 2:** Hazard ratios of continuous muscle and bone parameters in sex‐specific SD decrease for risk of second fracture

Muscle and bone parameters	Original analyses (45 vs. 221)				Competing risk analyses^a^ (45 vs. 249)			
	Unadjusted		Adjusted^b^		Unadjusted		Adjusted^b^	
	HR (95% CI)	*P* value	HR (95% CI)	*P* value	HR (95% CI)	*P* value	HR (95% CI)	*P* value
G.MaxM area (cm^2^)	1.64 (1.19, 2.25)	<0.01	1.32 (0.91, 1.91)	0.14	1.54 (1.15, 2.08)	<0.01	1.27 (0.89, 1.80)	0.18
G.MaxM density (HU)	1.85 (1.36, 2.51)	<0.01	1.49 (1.06, 2.10)	0.02	1.74 (1.27, 2.37)	<0.01	1.43 (0.96, 2.12)	0.08
G.Med/MinM density (HU)	1.98 (1.47, 2.67)	<0.01	1.68 (1.20, 2.35)	<0.01	1.84 (1.42, 2.39)	<0.01	1.61 (1.16, 2.23)	<0.01
FN CortThick (mm)	1.07 (0.78, 1.49)	0.67	1.08 (0.76, 1.53)	0.68	1.07 (0.77, 1.49)	0.68	1.07 (0.74, 1.54)	0.72
TH aBMD (g/cm^2^)	1.80 (1.34, 2.42)	<0.01	1.60 (1.11, 2.31)	0.01	1.72 (1.32, 2.25)	<0.01	1.50 (1.09, 2.08)	0.01
FN aBMD (g/cm^2^)	1.38 (1.01, 1.87)	0.04	1.19 (0.86, 1.66)	0.30	1.35 (0.94, 1.94)	0.11	1.14 (0.78, 1.67)	0.50
TR aBMD (g/cm^2^)	1.81 (1.30, 2.52)	<0.01	1.48 (1.01, 2.17)	0.04	1.71 (1.25, 2.34)	<0.01	1.40 (0.97, 2.01)	0.07
IT aBMD (g/cm^2^)	1.77 (1.33, 2.36)	<0.01	1.62 (1.13, 2.31)	0.01	1.71 (1.32, 2.22)	<0.01	1.53 (1.13, 2.06)	0.01
bCSA (cm^2^)	1.59 (1.17, 2.15)	<0.01	1.23 (0.88, 1.72)	0.22	1.54 (1.19, 1.98)	<0.01	1.19 (0.89, 1.58)	0.25
ACT (cm)	1.49 (1.09, 2.04)	0.01	1.18 (0.83, 1.67)	0.37	1.45 (1.08, 1.93)	0.01	1.13 (0.80, 1.61)	0.49
CSMI (cm^4^)	1.35 (0.98, 1.85)	0.06	1.14 (0.83, 1.56)	0.41	1.35 (1.00, 1.82)	0.05	1.14 (0.89, 1.46)	0.32
Z (cm^3^)	1.28 (0.98, 1.66)	0.07	1.13 (0.83, 1.55)	0.42	1.28 (0.99, 1.65)	0.06	1.12 (0.86, 1.47)	0.40
BR	0.74 (0.56, 0.98)	0.04	0.87 (0.64, 1.18)	0.36	0.78 (0.60, 1.02)	0.07	0.92 (0.66, 1.28)	0.61

BMD, bone mineral density; CI, confidence interval; HR, hazard ratio; SD, standard deviance.

^a^ As for refracture risk, we did the competing risk analyses using cause‐specific hazard models given that total deaths (*n* = 28) occurring in the absence of refracture events are the competing risks. The Fine–Gray model was further adopted for analysing competing risks as sensitivity analyses.

^b^ Adjusted for age, sex, T2DM, and Parker Mobility Score prior to first hip fracture surgery.

**Figure 3:**
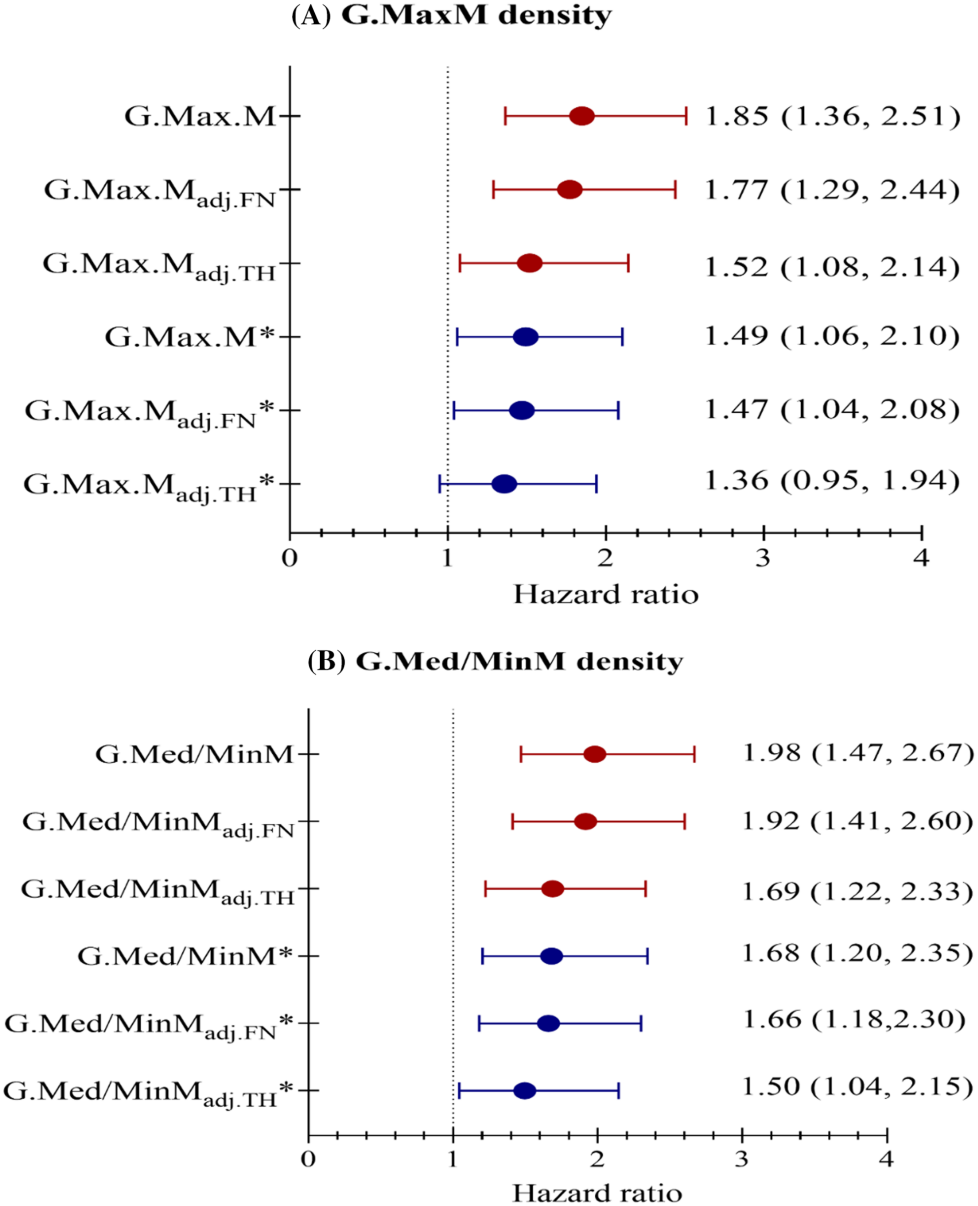
Unadjusted and adjusted hazard ratios of second hip fracture per one SD increase of G.MaxM density (*A*) and G.Med/MinM density (*B*). *Adjusted for age, sex, T2DM, and Parker Mobility Score prior to first hip fracture; SD, standard deviation; adj. FN refers to adjusted for femoral neck (FN) aBMD; adj.TH refers to adjusted for total hip (TH) aBMD.

*Figure* *3* shows unadjusted and adjusted hazard ratios of second hip fracture for G.MaxM (A) and G.Med/MinM densities (B). After adjustment for aBMD, HRs were attenuated slightly for density of G.MaxM (HR, 1.77; 95% CI, 1.29–2.44) and of G.Med/MinM (HR, 1.92; 95% CI, 1.41–2.60). Additional adjustments for age, sex, T2DM, and Parker Mobility Score further decreased the hazard ratios, but they remained significant (G.Med/MinM density: HR, 1.66; 95% CI, 1.18–2.30 when adjusted to FNaBMD; HR, 1.50; 95% CI, 1.04–2.15 when adjusted to THaBMD) with the exception of G.MaxM adjusted to TH aBMD.

### Muscle and bone: associations with first and second hip fracture

Univariate odds ratios comparing associations of no fracture vs. first fracture and of first vs. second fracture are shown in *Table* *S3*. With the exception of cross‐sectional moment of inertia, adjusted odds ratios for first hip fracture were significant for all muscle, aBMD, and structural parameters assessed in the study. However, this was not the case for OR of second fracture after first fracture. These were smaller by a factor of 3 to 4 and only significant for G.MaxM and G.Med/MinM densities, and TH, TR, and IT but not FN aBMD.

The AUC results are shown in *Table* *3*. Only AUCs of G.Med/MinM density, TH aBMD, and IT aBMD exceeded 70%, but no AUC value was higher than 75%. Several selected models combining muscle and BMD parameters were assessed, but the predictive ability did not increase beyond 75%.

**Table 3:** AUCs and 95% CI of first or second fracture per SD decrease in various continuous muscle and BMD parameters

Muscle and BMD parameters	Second fracture vs. non‐second fracture (45 vs. 221)				First fracture vs. never fracture (294 vs. 301)			
	Unadjusted model		Adjusted model^a^		Unadjusted model		Adjusted model^c^	
	AUC (95% CI)	*P* value^b^	AUC (95% CI)	*P* value^b^	AUC (95% CI)	*P* value^b^	AUC (95% CI)	*P* value^b^
Muscle models								
① G.MaxM area (cm^2^)	0.632 (0.546, 0.719)	0.65	0.731 (0.654, 0.808)	0.88	0.739 (0.699, 0.779)	0.55	0.776 (0.739, 0.814)	0.01
② G.MaxM density (HU)	0.654 (0.568, 0.741)	0.46	0.747 (0.673, 0.820)	0.40	0.831 (0.799, 0.863)	0.43	0.840 (0.808, 0.871)	0.89
③ G.Med/MinM density (HU)	0.690 (0.611, 0.770)	0.95	0.766 (0.697, 0.836)	0.42	0.936 (0.918, 0.955)	0.72	0.938 (0.920, 0.956)	0.99
① + ② + ③	0.690 (0.612, 0.768)	0.78	0.768 (0.699, 0.837)	0.87	0.938 (0.919, 0.956)	0.80	0.943 (0.926, 0.960)	0.94
② + ③	0.685 (0.604, 0.766)	0.67	0.768 (0.699, 0.837)	0.87	0.937 (0.919, 0.956)	0.63	0.940 (0.922, 0.957)	1.00
① + ③	0.694 (0.617, 0.771)	0.89	0.767 (0.697, 0.836)	0.42	0.938 (0.919, 0.956)	0.80	0.941 (0.924, 0.959)	0.81
BMD models								
④ TH aBMD (g/cm^2^)	0.670 (0.587, 0.753)	0.31	0.754 (0.682, 0.826)	0.56	0.830 (0.798, 0.862)	0.86	0.864 (0.835, 0.893)	0.94
⑤ FN aBMD (g/cm^2^)	0.600 (0.506, 0.693)	0.44	0.732 (0.656, 0.809)	0.60	0.834 (0.802, 0.866)	0.23	0.873 (0.845, 0.901)	0.18
⑥ TR aBMD (g/cm^2^)	0.655 (0.568, 0.742)	0.42	0.744 (0.672, 0.817)	0.90	0.849 (0.819, 0.880)	0.44	0.864 (0.835, 0.893)	0.49
⑦ IT aBMD (g/cm^2^)	0.675 (0.593, 0.757)	1.00	0.755 (0.682, 0.828)	0.75	0.824 (0.792, 0.857)	0.33	0.866 (0.837, 0.894)	0.99
④ + ⑤ + ⑥ + ⑦	0.674 (0.592, 0.756)	0.99	0.757 (0.685, 0.829)	0.50	0.875 (0.848, 0.903)	0.76	0.900 (0.876, 0.924)	0.83
④ + ⑤	0.671 (0.588, 0.753)	0.66	0.758 (0.686, 0.829)	0.70	0.846 (0.815, 0.877)	0.58	0.883 (0.856, 0.910)	0.72
④ + ⑥	0.671 (0.588, 0.753)	0.80	0.757 (0.685, 0.829)	0.27	0.849 (0.818, 0.879)	0.64	0.869 (0.840, 0.897)	0.30
④ + ⑦	0.674 (0.592, 0.756)	0.96	0.755 (0.682, 0.828)	0.75	0.830 (0.798, 0.862)	0.75	0.866 (0.838, 0.895)	0.99
Muscle and BMD models								
① + ③ + ④ + ⑤	0.732 (0.664, 0.800)	0.11	0.781 (0.715, 0.847)	0.75	0.961 (0.947, 0.975)	0.92	0.965 (0.952, 0.978)	1.00
③ + ④	0.733 (0.665, 0.800)	0.16	0.779 (0.712, 0.845)	0.75	0.955 (0.941, 0.970)	0.47	0.959 (0.945, 0.973)	0.57
③ + ⑦	0.733 (0.664, 0.803)	0.30	0.778 (0.712, 0.845)	0.78	0.956 (0.942, 0.971)	0.99	0.961 (0.947, 0.974)	0.50

AUC, area under the receiver operating characteristic curve; BMD, bone mineral density; CI, confidence interval.

^a^ Adjusted for age, sex, type 2 diabetes, and Parker Mobility Score prior to first hip fracture surgery.

^b^ *P* value from the Hosmer–Lemeshow tests.

^c^ Adjusted for age, sex, and type 2 diabetes.

## Discussion

This is the first study summarizing the strength of bone and muscle parameters for the prediction of second hip fracture. According to current osteoporosis guidelines,^13^, ^25^, ^26^ all low‐trauma hip fracture patients should receive second hip fracture prevention treatment, but the prevalence of a lack of proper medical management remains high.^2^, ^13^ Therefore, it is important to identify patients at high risk of a second hip fracture. Apart from acute medical management and surgery, physicians and orthopaedic surgeons should also assess the risk of future hip fracture and council patients on an effective prevention strategy, mostly involving osteoporosis medication.

Almost all osteoporotic hip fractures are related to falls, and the risk of falls is in part determined by muscle function and quality. Many studies have shown a high association between muscle quality assessments and hip fracture risk. For example, our previous study showed that muscle density measured with CT in Hounsfield units was more strongly associated with acute hip fracture than muscle size or aBMD.^18^ The clinical significance of parameters characterizing muscle quality was also observed in other studies.^15^, ^17^, ^27^, ^28^

Interestingly, the relevance of muscle assessments for second hip fracture prediction has not been reported, although it is well known that the first low‐energy hip fracture has detrimental effects on BMD and muscle performance. Our results indicated that G.Med/MinM density and IT aBMD were the most relevant parameters in predicting second hip fracture. After adjusting for TH or FN aBMD, associations with second hip fracture remained significant for G.Med/MinM density but not for G.Max density (*Figure* *3*). The G.med/min muscle, referred to as the ‘rotator cuff of the hip’, plays an important role in gait stability. It inserts on the greater TR of the femur and serves as the major abductor and rotator of the hip during normal gait to maintain balance. Our outcomes highlight the G.med/min muscle as a potential target for future interventional approaches.

Interestingly, FN aBMD was not a significant predictor of second hip fracture risk although our study showed that FN aBMD was indeed a strong predictor of first hip fracture, in accordance with the use of FN aBMD in the Fracture Risk Assessment Tool (FRAX) for 10 year hip fracture risk assessments.^29^ In contrast, TR aBMD was a significant predictor of second hip fracture, confirming the findings of a previous biomechanical study in which Cheng *et al*. reported that TR aBMD was more strongly associated with proximal femur strength than FN aBMD.^30^ In another study, TR volumetric BMD (vBMD) in combination with cortical thickness measured by quantitative CT, but not FN vBMD, was significantly associated with acute femoral fracture, while FN vBMD alone showed the highest association with FN fractures and TR vBMD alone with trochanteric fractures.^31^ In conclusion, our results for FN aBMD and TR aBMD may in part be related to the site of the FN fracture. Second hip fractures were mostly trochanteric fractures while the first hip fractures were mostly fractures of the neck. Whether this finding was specific for our study or whether second hip fractures in general are mostly trochanteric fractures still has to be shown, but our observations indicate that special attention should be given to bone density of the trochanteric region for the prevention of second hip fracture.

This is also the first study to compare the ability of bone and muscle measures to discriminate second hip fracture vs. first hip fracture and first hip fracture vs. no fracture. Despite the excellent discrimination of a first hip fracture by the combined muscle and bone models (AUC 0.959–0.965), the performance for discrimination of second hip fracture was much poorer. Results for discriminating first hip fracture risk were similar to our previous report (AUC 0.923–0.958) in a propensity score matching case–control study.^18^ Therefore, we hypothesize that the risk of second hip fracture is more related to falls and not so much to bone strength and that the risk of falls is also highly impacted by other conditions such as neurologic function and impairment of sight.

Strengths of our study are that all 302 hip fracture patients included in the analysis had low‐energy fractures, and all patients with incident hip fractures were scanned within 48 h, which minimizes fracture‐related changes of bone and muscle tissues. Another strength was the availability of a relatively healthy control group to compare the performance of bone and muscle parameters and related models in predicting first and second hip fractures. Finally, we used the Parker Mobility Score to exclude those hip fracture patients with impaired mobility as we hypothesized that hip fracture patients with poor or no mobility had a low risk of second hip fracture.

This study has several limitations. There were no follow‐up measurements in the control group, and therefore, a prediction of first fracture was not possible, but pooling the groups from the CASH and CSHFE studies provided the possibility to compare fracture discrimination. Follow‐up of the CSHFE subjects was performed after 4.5 years to ensure a high cumulative incidence of second hip fracture, but earlier follow‐up measurements were not available. Third, CT scans of the hip were performed at baseline and were not repeated, so data on changes of bone and muscle parameters after first hip fracture are lacking. Fourth, we did not perform a detailed assessment of physical function, which may have added to a better understanding of the causes of second hip fracture, in particular relating to falls. However, we did use the Parker Mobility Score to exclude patients with poor mobility.

In conclusion, muscle density is an independent predictor for risk of first and second hip fracture. The ability of BMD and muscle parameters to predict second hip fracture is significantly lower than for first hip fracture, suggesting a different causal mechanism for second hip fractures more related to falls. This could direct a stronger focus on the prevention of falls in the second prevention of hip fracture patients. In addition, our results emphasize the importance of trochanteric aBMD over FN aBMD for the prediction of second hip fracture. Together, our results may lead to a paradigm shift in the assessment of second hip fracture risk and prevention.

## Conflict of interest

K.E. is a part‐time employee of BioClinica, Inc. Other authors declare no conflict of interest.

## Funding

This work is supported in part by the National Natural Science Foundation of China (Grant 81901718 and 81771831), the Beijing Natural Science Foundation–Haidian Primitive Innovation Joint Fund (Grant L172019), and Beijing Hospitals Authority Clinical Medicine Development of Special Funding Support (code: ZYLX202107).

## Supporting information


**Figure S1** A representative case with second hip fracture. (A) Measurement of cross‐sectional area and mean computed tomography values of the gluteus maximus muscle at the level of the greater trochanter of the femur. (B) Measurement of the gluteus medius and minimus muscle at the third sacral level. Muscle region is represented by the area highlighted in red. (C) Regions of interest (ROIs) analyzed in the proximal femur by QCTPro CTXA. (D) Femoral neck cortical thickness measured by MIAF Femur.
**Figure S2** Distribution of first hip fracture type among the patients. FN, femoral neck; TR, trochanter.
**Table S1** Hazard ratios of various continuous muscle and bone parameters in SD decrease for refracture risk.
**Table S2** Hazard ratios of continuous muscle and bone parameters for further adjustment in sex‐specific SD decrease for second hip fracture risk.
**Table S3** Odds ratios of continuous muscle and bone parameters in sex‐specific SD decrease for fracture risk.
