# Supplementary material for: Muscle density is an independent risk factor of second hip fracture: a prospective cohort study
Source: J Cachexia Sarcopenia Muscle. 2022 Apr 15;13(3):1927–37. doi: 10.1002/jcsm.12996 (PMC9178374; doi:10.1002/jcsm.12996)
Supplement: Supplementary file 1 — Figure S1 A representative case with second hip fracture. (A) Measurement of cross‐sectional area and mean computed tomography values of the gluteus maximus muscle at the level of the greater trochanter of the femur. (B) Measurement of the gluteus medius and minimus muscle at the third sacral level. Muscle region is represented by the area highlighted in red. (C) Regions of interest (ROIs) analyzed in the proximal femur by QCTPro CTXA. (D) Femoral neck cortical thickness measured by MIAF Femur. Figure S2 Distribution of first hip fracture type among the patients. FN, femoral neck; TR, trochanter. Table S1 Hazard ratios of various continuous muscle and bone parameters in SD decrease for refracture risk. Table S2 Hazard ratios of continuous muscle and bone parameters for further adjustment in sex‐specific SD decrease for second hip fracture risk. Table S3 Odds ratios of continuous muscle and bone parameters in sex‐specific SD decrease for fracture risk. [file JCSM-13-1927-s001.docx]

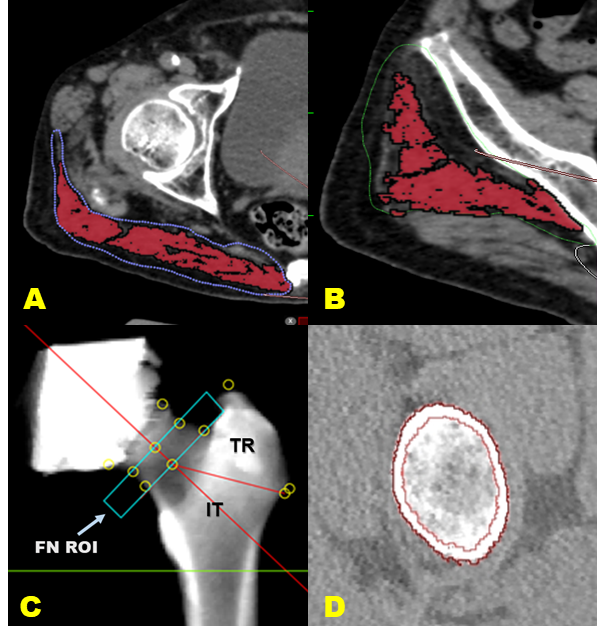


**Supplementary Figure S1 A representative case with second hip fracture.** (A) Measurement of cross-sectional area and mean computed tomography values of the gluteus maximus muscle at the level of the greater trochanter of the femur. (B) Measurement of the gluteus medius and minimus muscle at the third sacral level. Muscle region is represented by the area highlighted in red. (C) Regions of interest (ROIs) analyzed in the proximal femur by QCTPro CTXA. (D) Femoral neck cortical thickness measured by MIAF Femur.

**
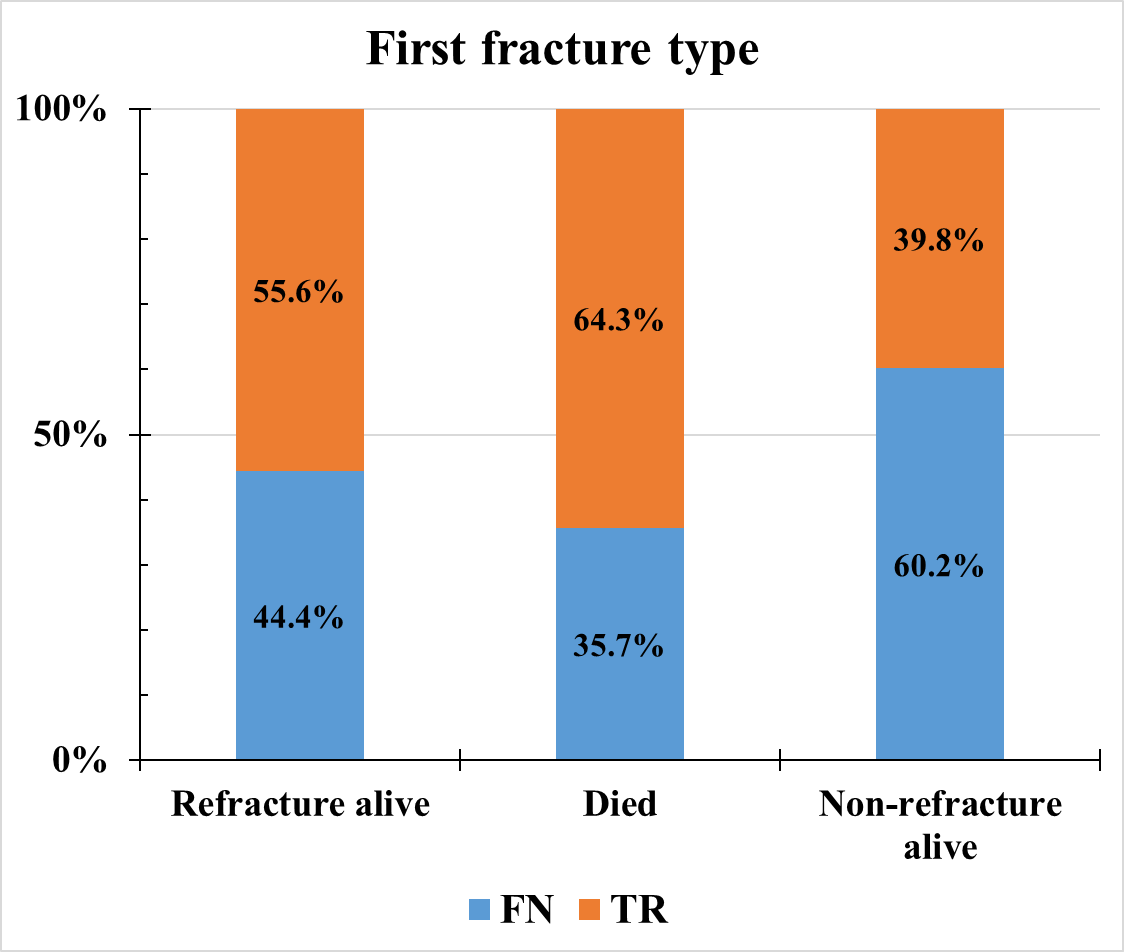
**

**Supplementary Figure S2 Distribution of first hip fracture type among the patients. FN, femoral neck; TR, trochanter.**

**Supplementary Table S1. Hazard ratios of various continuous muscle and bone parameters in SD decrease for refracture risk**

| **Muscle and bone parameters** | **Original analyses (45 vs. 257)** | | | | |  | **Original analyses (45 vs. 371)** | | | | |
| --- | --- | --- | --- | --- | --- | --- | --- | --- | --- | --- | --- |
|  | **Unadjusted** | |  | **Adjusted^1^** | |  | **Unadjusted** | |  | **Adjusted^1^** | |
|  | **HR (95% CI)** | **P value** |  | **HR (95% CI)** | **P value** |  | **HR (95% CI)** | **P value** |  | **HR (95% CI)** | **P value** |
| G.MaxM area (cm^2^) | 1.59 (1.16, 2.19) | <0.01 |  | 1.41 (0.99, 2.03) | 0.06 |  | 1.49 (1.09, 2.05) | 0.01 |  | 1.38 (0.96, 1.99) | 0.09 |
| G.MaxM density (HU) | 1.92 (1.42, 2.59) | <0.01 |  | 1.71 (1.21, 2.41) | <0.01 |  | 1.81 (1.35, 2.44) | <0.01 |  | 1.64 (1.17, 2.31) | <0.01 |
| G.Med/MinM density (HU) | 2.04 (1.52, 2.75) | <0.01 |  | 1.90 (1.37, 2.62) | <0.01 |  | 1.95 (1.44, 2.63) | <0.01 |  | 1.86 (1.35, 2.57) | <0.01 |
| FN CortThick (mm) | 1.02 (0.74, 1.41) | 0.91 |  | 1.00 (0.71, 1.40) | 0.99 |  | 1.03 (0.74, 1.43) | 0.86 |  | 1.03 (0.72, 1.45) | 0.89 |
| TH aBMD (g/cm^2^) | 1.70 (1.27, 2.27) | <0.01 |  | 1.56 (1.07, 2.28) | 0.02 |  | 1.67 (1.25, 2.23) | <0.01 |  | 1.58 (1.08, 2.30) | 0.02 |
| FN aBMD (g/cm^2^) | 1.36 (1.01, 1.84) | 0.05 |  | 1.18 (0.85, 1.65) | 0.33 |  | 1.34 (0.99, 1.80) | 0.06 |  | 1.15 (0.83, 1.60) | 0.40 |
| TR aBMD (g/cm^2^) | 1.73 (1.25, 2.41) | <0.01 |  | 1.45 (0.98, 2.15) | 0.07 |  | 1.67 (1.21, 2.31) | <0.01 |  | 1.46 (0.99, 2.15) | 0.05 |
| IT aBMD (g/cm^2^) | 1.66 (1.25, 2.21) | <0.01 |  | 1.57 (1.08, 2.27) | 0.02 |  | 1.63 (1.23, 2.15) | <0.01 |  | 1.54 (1.08, 2.19) | 0.02 |
| bCSA (cm^2^) | 1.48 (1.10, 1.99) | 0.01 |  | 1.19 (0.85, 1.65) | 0.31 |  | 1.44 (1.08, 1.94) | 0.01 |  | 1.20 (0.86, 1.67) | 0.29 |
| ACT (cm) | 1.32 (0.98, 1.78) | 0.07 |  | 1.03 (0.73, 1.46) | 0.85 |  | 1.30 (0.97, 1.75) | 0.08 |  | 1.06 (0.75, 1.48) | 0.75 |
| CSMI (cm^4^) | 1.32 (0.97, 1.78) | 0.07 |  | 1.15 (0.85, 1.56) | 0.36 |  | 1.32 (0.97, 1.79) | 0.08 |  | 1.15 (0.83, 1.57) | 0.40 |
| Z (cm^3^) | 1.24 (0.97, 1.59) | 0.09 |  | 1.11 (0.83, 1.47) | 0.49 |  | 1.24 (0.97, 1.60) | 0.09 |  | 1.09 (0.81, 1.47) | 0.56 |
| BR | 0.87 (0.66, 1.17) | 0.36 |  | 1.02 (0.75, 1.38) | 0.90 |  | 0.90 (0.68, 1.20) | 0.48 |  | 1.01 (0.75, 1.36) | 0.97 |

Note: SD, standard deviance; HR, hazard ratio; CI, confidence interval; BMD, bone mineral density; G.MaxM, gluteus maximus muscle; G.Med/MinM, gluteus medius and minimus muscle; FN CortThick, Cortical thickness of femoral neck; aBMD, areal bone mineral density; TH, total hip; FN, femoral neck; TR, trochanter; IT, intertrochanter; bCSA, bone cross-sectional area; ACT, average cortical thickness; CSMI, cross-sectional moment of inertia; Z, section modulus; BR, buckling ratio.

^1^ Adjusted for age, sex, T2DM and Parker Mobility Score prior to first hip fracture surgery.

**Supplementary Table S2 Hazard ratios of continuous muscle and bone parameters for further adjustment in sex-specific SD decrease for second hip fracture risk**

| **Muscle and bone parameters** | **Adjusted^1^(Original analyses)** | |  | **Adjusted^1^ (Competing risk analyses)** | |
| --- | --- | --- | --- | --- | --- |
|  | **HR (95% CI)** | **P value** |  | **HR (95% CI)** | **P value** |
| G.MaxM area (cm^2^) | 1.30 (0.90, 1.88) | 0.16 |  | 1.24 (0.87, 1.76) | 0.23 |
| G.MaxM density (HU) | 1.47 (1.04, 2.08) | 0.03 |  | 1.42 (0.96, 2.10) | 0.08 |
| G.Med/MinM density (HU) | 1.72 (1.23, 2.40) | <0.01 |  | 1.64 (1.18, 2.28) | <0.01 |
| FN CortThick (mm) | 1.04 (0.74, 1.48) | 0.81 |  | 1.04 (0.74, 1.46) | 0.81 |
| TH aBMD (g/cm^2^) | 1.70 (1.17, 2.47) | 0.01 |  | 1.54 (1.11, 2.14) | 0.01 |
| FN aBMD (g/cm^2^) | 1.20 (0.86, 1.69) | 0.29 |  | 1.15 (0.78, 1.69) | 0.49 |
| TR aBMD (g/cm^2^) | 1.53 (1.04, 2.25) | 0.03 |  | 1.41 (0.98, 2.03) | 0.06 |
| IT aBMD (g/cm^2^) | 1.71 (1.19, 2.45) | <0.01 |  | 1.56 (1.16, 2.11) | <0.01 |
| bCSA (cm^2^) | 1.29 (0.91, 1.83) | 0.15 |  | 1.24 (0.90, 1.70) | 0.18 |
| ACT (cm) | 1.21 (0.84, 1.74) | 0.31 |  | 1.16 (0.80, 1.68) | 0.44 |
| CSMI (cm^4^) | 1.16 (0.85, 1.60) | 0.35 |  | 1.16 (0.89, 1.51) | 0.28 |
| Z (cm^3^) | 1.16 (0.85, 1.58) | 0.35 |  | 1.15 (0.87, 1.51) | 0.34 |
| BR | 0.83 (0.61, 1.13) | 0.24 |  | 0.89 (0.64, 1.23) | 0.47 |

Note: ^1^Adjusted for age, sex, hypertension, previous fracture, osteoarthritis, coronary heart disease, type 2 diabetes and Parker Mobility Score prior to first hip fracture surgery.

**Supplementary Table S3. Odds ratios of continuous muscle and bone parameters in sex-specific SD decrease for fracture risk**

| **Muscle and bone parameters** | **Second fracture vs. first fracture (45 vs. 249)** | | | | |  | **First fracture vs. never fracture (294 vs. 301)** | | | | |
| --- | --- | --- | --- | --- | --- | --- | --- | --- | --- | --- | --- |
|  | **Unadjusted** | |  | **Adjusted^1^** | |  | **Unadjusted** | |  | **Adjusted^1^** | |
|  | **OR (95% CI)** | **P value** |  | **OR (95% CI)** | **P value** |  | **OR (95% CI)** | **P value** |  | **OR (95% CI)** | **P value** |
| G.MaxM area (cm^2^) | 1.55 (1.10, 2.19) | 0.01 |  | 1.26 (0.85, 1.88) | 0.25 |  | 2.78 (2.25, 3.43) | <0.01 |  | 2.29 (1.83, 2.86) | <0.01 |
| G.MaxM density (HU) | 1.73 (1.25, 2.40) | <0.01 |  | 1.45 (1.01, 2.06) | 0.04 |  | 4.79 (3.68, 6.23) | <0.01 |  | 4.51 (3.40, 5.99) | <0.01 |
| G.Med/MinM density (HU) | 1.98 (1.40, 2.78) | <0.01 |  | 1.79 (1.25, 2.57) | <0.01 |  | 24.80 (14.75, 41.70) | <0.01 |  | 28.97 (16.48, 50.94) | <0.01 |
| FN CortThick (mm) | 1.00 (0.71, 1.43) | 0.98 |  | 0.98 (0.67, 1.44) | 0.93 |  | 2.57 (2.03, 3.25) | <0.01 |  | 3.27 (2.45, 4.37) | <0.01 |
| TH aBMD (g/cm^2^) | 1.79 (1.30, 2.47) | <0.01 |  | 1.64 (1.11, 2.44) | 0.01 |  | 6.69 (4.91, 9.11) | <0.01 |  | 5.92 (4.31, 8.14) | <0.01 |
| FN aBMD (g/cm^2^) | 1.37 (1.00, 1.89) | 0.05 |  | 1.16 (0.82, 1.65) | 0.41 |  | 6.49 (4.78, 8.81) | <0.01 |  | 5.79 (4.24, 7.91) | <0.01 |
| TR aBMD (g/cm^2^) | 1.77 (1.25, 2.51) | <0.01 |  | 1.46 (0.98, 2.16) | 0.06 |  | 6.66 (4.89, 9.06) | <0.01 |  | 5.91 (4.29, 8.13) | <0.01 |
| IT aBMD (g/cm^2^) | 1.77 (1.3, 2.42) | <0.01 |  | 1.69 (1.14, 2.49) | 0.01 |  | 6.55 (4.82, 8.90) | <0.01 |  | 5.98 (4.34, 8.24) | <0.01 |
| bCSA (cm^2^) | 1.60 (1.15, 2.23) | 0.01 |  | 1.27 (0.88, 1.85) | 0.21 |  | 4.38 (3.38, 5.68) | <0.01 |  | 3.77 (2.88, 4.94) | <0.01 |
| ACT (cm) | 1.50 (1.08, 2.10) | 0.02 |  | 1.25 (0.86, 1.81) | 0.24 |  | 5.02 (3.80, 6.63) | <0.01 |  | 4.30 (3.24, 5.70) | <0.01 |
| CSMI (cm^4^) | 1.39 (0.99, 1.95) | 0.06 |  | 1.22 (0.83, 1.80) | 0.31 |  | 1.24 (1.06, 1.44) | 0.01 |  | 1.13 (0.95, 1.35) | 0.18 |
| Z (cm^3^) | 1.30 (0.98, 1.73) | 0.07 |  | 1.20 (0.83, 1.74) | 0.33 |  | 1.56 (1.32, 1.85) | <0.01 |  | 1.49 (1.23, 1.81) | <0.01 |
| BR | 0.77 (0.57, 1.03) | 0.08 |  | 0.83 (0.61, 1.14) | 0.24 |  | 0.24 (0.18, 0.31) | <0.01 |  | 0.27 (0.20, 0.35) | <0.01 |

Note: ^1^ Adjusted for age and sex
